# Neurodegenerative Disease Tauopathies

**DOI:** 10.1146/annurev-pathmechdis-051222-120750

**Published:** 2023-10-13

**Authors:** Benjamin C. Creekmore, Ryohei Watanabe, Edward B. Lee

**Affiliations:** Translational Neuropathology Research Laboratory, Department of Pathology and Laboratory Medicine, Perelman School of Medicine at the University of Pennsylvania, Philadelphia, Pennsylvania, USA;

**Keywords:** Alzheimer’s disease, AD, frontotemporal lobar degeneration, FTLD, microtubule-associated protein tau, MAPT, tauopathy, proteostasis, cryogenic electron microscopy, cryo-EM

## Abstract

Tauopathies are a diverse group of progressive and fatal neurodegenerative diseases characterized by aberrant tau inclusions in the central nervous system. Tau protein forms pathologic fibrillar aggregates that are typically closely associated with neuronal cell death, leading to varied clinical phenotypes including dementia, movement disorders, and motor neuron disease. In this review, we describe the clinicopathologic features of tauopathies and highlight recent advances in understanding the mechanisms that lead to spread of pathologic aggregates through interconnected neuronal pathways. The cell-to-cell propagation of tauopathy is then linked to posttranslational modifications, tau fibril structural variants, and the breakdown of cellular protein quality control.

## INTRODUCTION

Tau has been identified as the main component of insoluble fibrils that make up inclusions in a wide range of diseases—given the name tauopathies by Bernardino Ghetti and Michel Goedert (1). There are now dozens of diseases in an ever-growing list that have been associated with tau aggregates in the brain and/or spinal cord (1) of which the more widely recognized tauopathies are listed in Table 1. Alzheimer’s disease (AD) is by far the most common tauopathy, and the amount and spread of tau accumulation in the form of neurofibrillary tangles (NFTs), dystrophic neurites, and threads correlates well with the degree of cognitive impairment and dementia (2–5). A predominant feature of most of these diseases is brain atrophy due to neuron death. Understanding how tau aggregates and spreads through the brain, and how tau aberrations cause downstream neurotoxicity, may help guide effective therapeutic development. Currently there are only a small number of effective therapies for AD despite more than a century of investigation, and there are no therapies that directly target tau accumulation.

**Figure 1:**
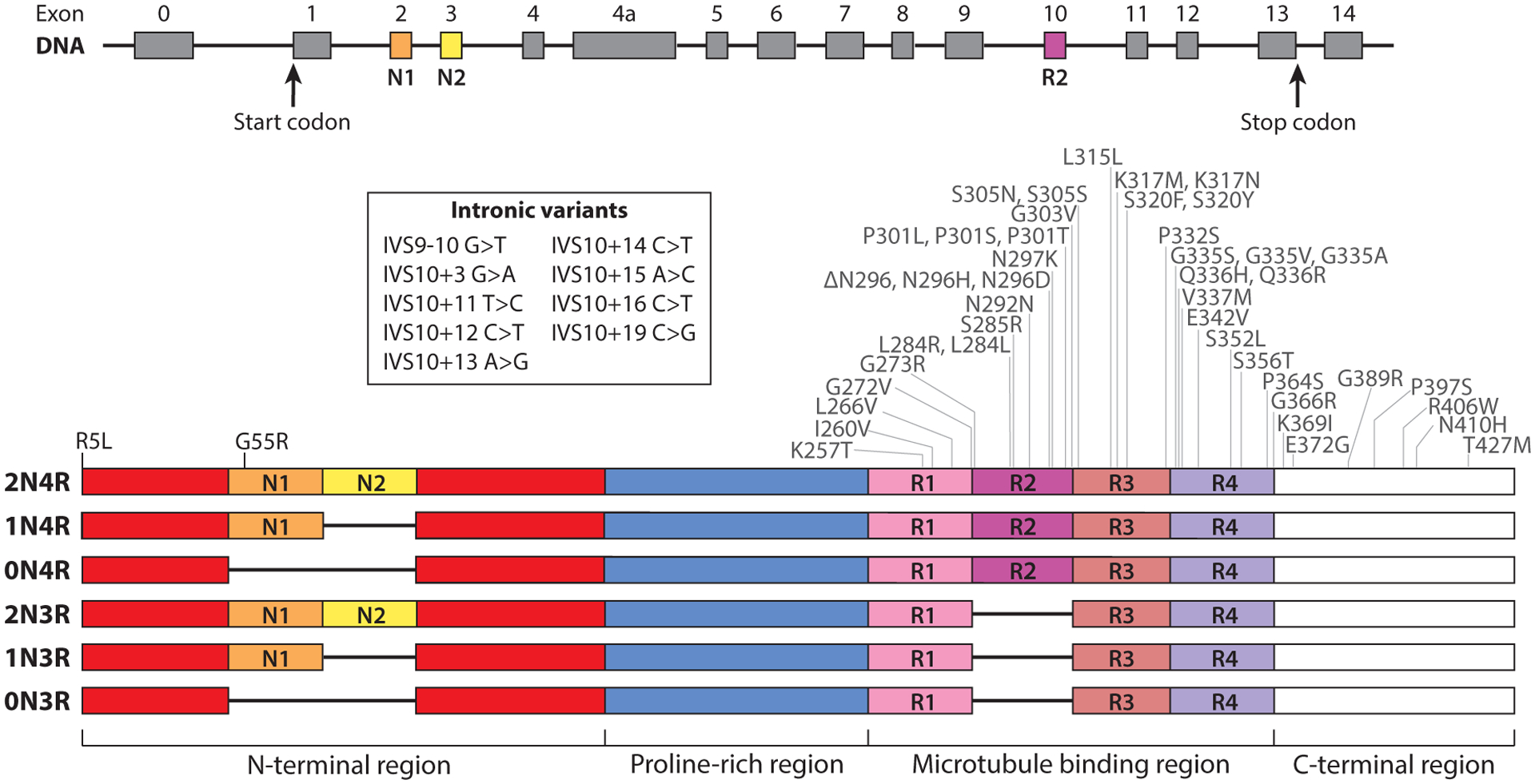
Pathogenic variants and major splicing variants of tau. Tau is encoded by the *MAPT* gene on chromosome 17 in humans that contains 16 exons of which exons 1, 4, 5, 7, 9, 11, 12, and 13 are constitutively included. There are six major isoforms in the human brain produced via alternative splicing (7). Splicing of exons 2 and 3 regulates the inclusion of 0, 1, or 2 N-terminal inserts (N1: *orange*, N2: *yellow*) into the N-terminal region (*red*). Alternative splicing of exon 10 (*dark pink*) creates isoforms with either three or four microtubule binding region (MTBR) repeats (R1: *light pink*, R2: *dark pink*, R3: *salmon*, R4: *purple*). Tau also has a proline-rich region (*blue*) and a C-terminal region (*white*). Some mutations in *MAPT* lead to pathogenic amino acid changes or alterations in the ratio of tau isoforms (133). Pathogenic intronic variants in *MAPT* are primarily found in intron 10 and affect the isoform ratio. Pathogenic exonic variants primarily cluster in the MTBR that is responsible for microtubule binding and makes up the tau fibril core.

Tau protein normally functions to stabilize microtubules in neuronal axons (6), and tau expression is normally low in glia. Tau is encoded by the *MAPT* gene on chromosome 17. There are six major isoforms in the human brain produced by alternative splicing (7). Splicing of exons 2 and 3 regulates inclusion of the amino-terminal insert (N) to create 0N (without exons 2 and 3), 1N (with only exon 2), and 2N (with both exons 2 and 3) (see Figure 1 for the primary structure of tau including spliced exons). Alternative splicing of exon 10 creates isoforms with either three microtubule binding regions (MTBRs) (3R, without exon 10) or four MTBRs (4R, with exon 10) (Figure 1). Therefore, tau protein length is between 352 (0N3R) and 441 (2N4R, also known as T40) amino acids. All six tau isoforms are expressed at roughly equal ratios in normal brain. However, the ratio of 3R and 4R tau has received attention in part because alterations in the expression of tau isoforms due to mis-splicing of exon 10 can lead to familial forms of tauopathy. 4R tau has a greater ability than 3R tau to stabilize microtubules, suggesting that the appropriate ratio of 3R/4R tau is required for normal function and indicating why the alteration of this ratio due to pathogenic *MAPT* variants may lead to disease (8). An additional form of tau, known as big tau, includes an additional exon, exon 4a, that adds more than 250 amino acids to the N-terminal region. Big tau is expressed mainly in the adult peripheral nervous system but is also expressed in the central nervous system, and little is known about how it affects the normal functions of tau or whether it may have a role in disease (9). Furthermore, the various tauopathies can be classified by the isoform present in inclusions, as different tauopathies are characterized by having either 3R, 4R, or 3R+4R tau inclusions (Table 1). Some tauopathies, such as chronic traumatic encephalopathy, can even include a mixture of different inclusion types, each consisting of distinct isoforms with neuronal aggregates containing 3R+4R tau adjacent to astrocytic aggregates containing only 4R tau (10).

Autosomal dominant pathogenic variants of only two different genes have been identified to cause primary tauopathies. Pathogenic variants in the *MAPT* gene itself cause frontotemporal dementia (FTD), sometimes with parkinsonism, and primarily occur in exons 9–13 that make up the MTBR (Figure 1). Many of these variants are thought to disrupt tau’s ability to interact with microtubules (11), while some pathogenic *MAPT* variants, including intronic variants, affect the ratio of 3R and 4R tau and lead to tau inclusions with primarily 3R versus 4R tau (12). More recently, we identified a pathogenic variant in valosin-containing protein (*VCP*) that causes a previously undescribed form of FTD called vacuolar tauopathy, characterized by the accumulation of AD-like NFTs and abnormal neuronal vacuoles (13).

Other autosomal dominant diseases have also been associated with tauopathy, albeit together with other pathologies. For example, pathogenic variants in *APP* [precursor to amyloid-β (Aβ) peptide] and *PSEN1/PSEN2* (subunit of the protease that generates Aβ peptide) lead to the over-production and/or altered production of Aβ peptide, resulting in most instances in an early-onset AD clinical phenotype and the presence of AD neuropathologic change in the brain consisting of Aβ plaques and tau-positive NFTs. Though autosomal dominant causes of tauopathy represent a very small fraction of all tauopathies, they may provide insight into the underlying mechanisms of sporadic tauopathies.

Beyond Mendelian forms of dementia, there have been many genes identified as potential risk factors for tauopathies, mostly AD, through genome-wide association studies (GWAS). For example, single-nucleotide polymorphisms in *TREM2* confer increased risk for AD (14), and human neuropathology studies have shown that these *TREM2* risk variants are associated with several changes in the brain including reduced numbers of microglia per amyloid plaque (15, 16) and a change in the distributional pattern of disease (17). Individuals with *TREM2* risk variants were more likely to have atypical nonamnestic AD clinical phenotypes, which correlated with a higher proportion of cases with an atypical, hippocampal sparing distribution of NFTs (17). Other genetic risk variants, such as those associated with *BIN1* and *APOE*, are also likely to influence human tauopathy.

**Figure 2:**
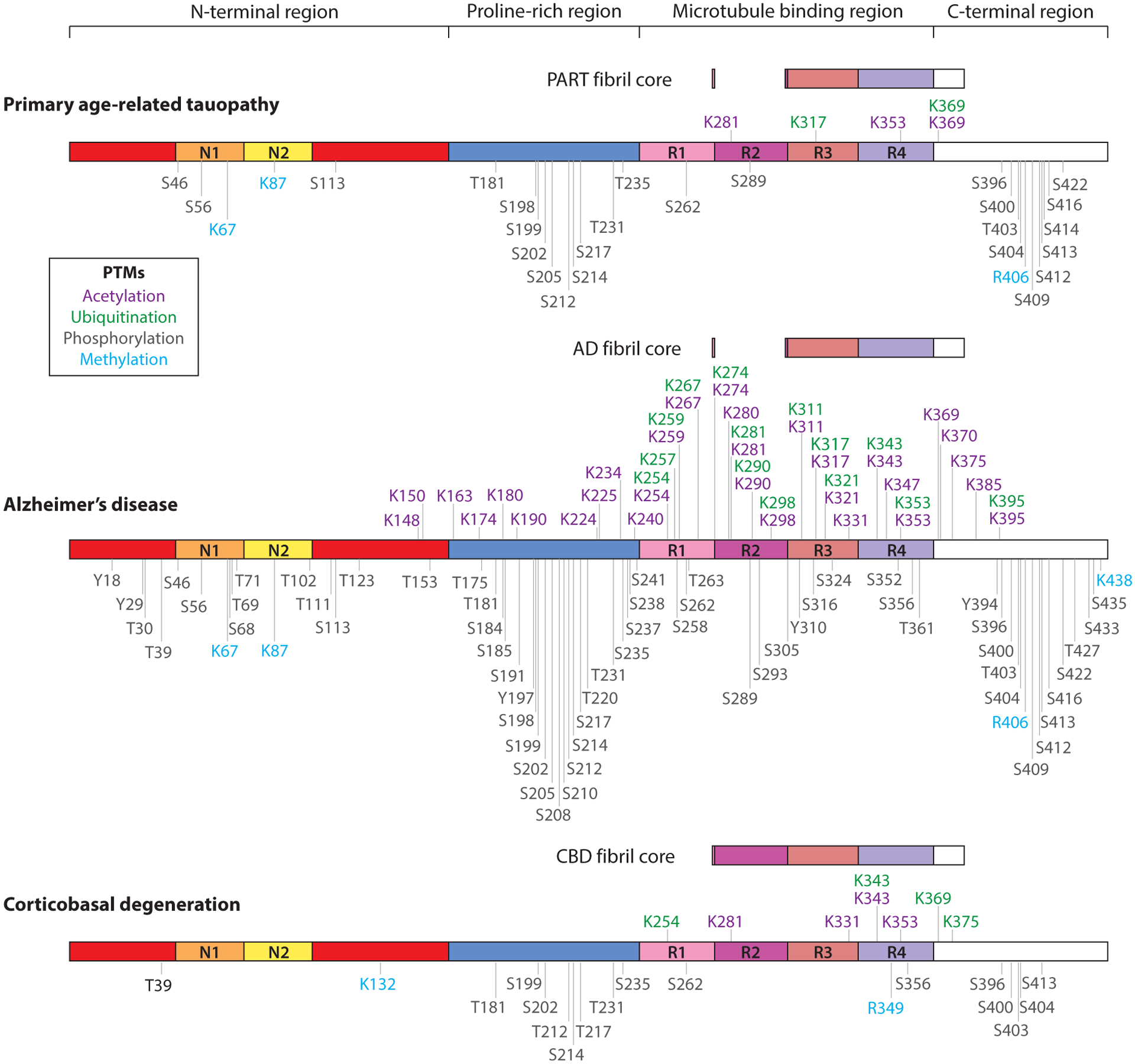
Posttranslational modifications (PTMs) of disease-relevant tau fibrils. Sarkosyl-insoluble tau fibrils are found in cognitively normal individuals with primary age-related tauopathy (PART) and cognitively impaired individuals with Alzheimer’s disease (AD) or corticobasal degeneration (CBD). The core of these fibrils is made of part of the microtubule binding region (*pink*/*purple*) and C-terminal region (*white*). These different fibrils are variably modified by PTMs including acetylation (*purple*), ubiquitination (*green*), phosphorylation (*dark gray*), and methylation (*light blue*) (20, 22).

Tau protein is subject to numerous posttranslational modifications (PTMs) (18). Some PTMs such as phosphorylation regulate tau’s normal function in cells. Thus, while normal tau protein is phosphorylated at multiple sites, these can often be difficult to detect in autopsied brain tissue because these phosphoepitopes are lost over time ex vivo (19). However, many PTMs, including acetylation, phosphorylation, and ubiquitination, are altered in disease-associated tau fibrils (Figure 2) and have been proposed to help initiate or stabilize fibrillization of tau (20–22). Markedly increased and more stable phosphorylation at these epitopes has long been associated with alterations in tauopathies and has been used frequently as a marker of pathologic change (23). Phosphorylation has been proposed to be one of the first alterations to tau in disease pathogenesis in that phosphorylation leads to disruption of tau’s ability to associate with microtubules and reduces tau’s solubility, rendering it more prone to aggregation (18, 24). Moreover, phosphorylation patterns are not static but appear to change as tau aggregates mature in the human brain. As NFTs mature from pretangles to mature tangles to ghost tangles, different phosphoepitopes appear to be enriched in tau aggregates that have been proposed to represent inclusions of different stages of maturity. For example, phosphoepitopes at S202 and T205 are prominent at the pretangle stage while S396 and S404 are prominent at the mature tangle stage, with neither being prominent in ghost tangles (25). In contrast, MN423, an antibody raised against pronase-treated tau preparations that recognizes an E391 truncated tau epitope, primarily detects ghost tangles.

While tau phosphorylation remains the best studied tau PTM, other alterations have been garnering more attention. For example, acetylation has more recently been shown to disrupt tau’s ability to be degraded and may further contribute to insolubility of tau later in disease than phosphorylation (22, 26–28). Ubiquitination of tau has become an interest in terms of disease pathogenesis as it has generally been thought of as beneficial for clearance of tau due to its role in directing proteins to the proteosome or autophagosomes for proteolysis. Recent data may suggest that ubiquitination may be paradoxically contributing to disease progression or at least not efficiently assisting in tau clearance (20, 22, 29). There are likely additional complex interactions as well, as tau acetylation has been reported to inhibit and compete with tau ubiquitination (30). The complex interplay of tau and its variable PTMs likely has a role in the underlying mechanism of the neurotoxicity of tau.

## CLINICOPATHOLOGIC FINDINGS IN TAUOPATHIES

**Figure 3:**
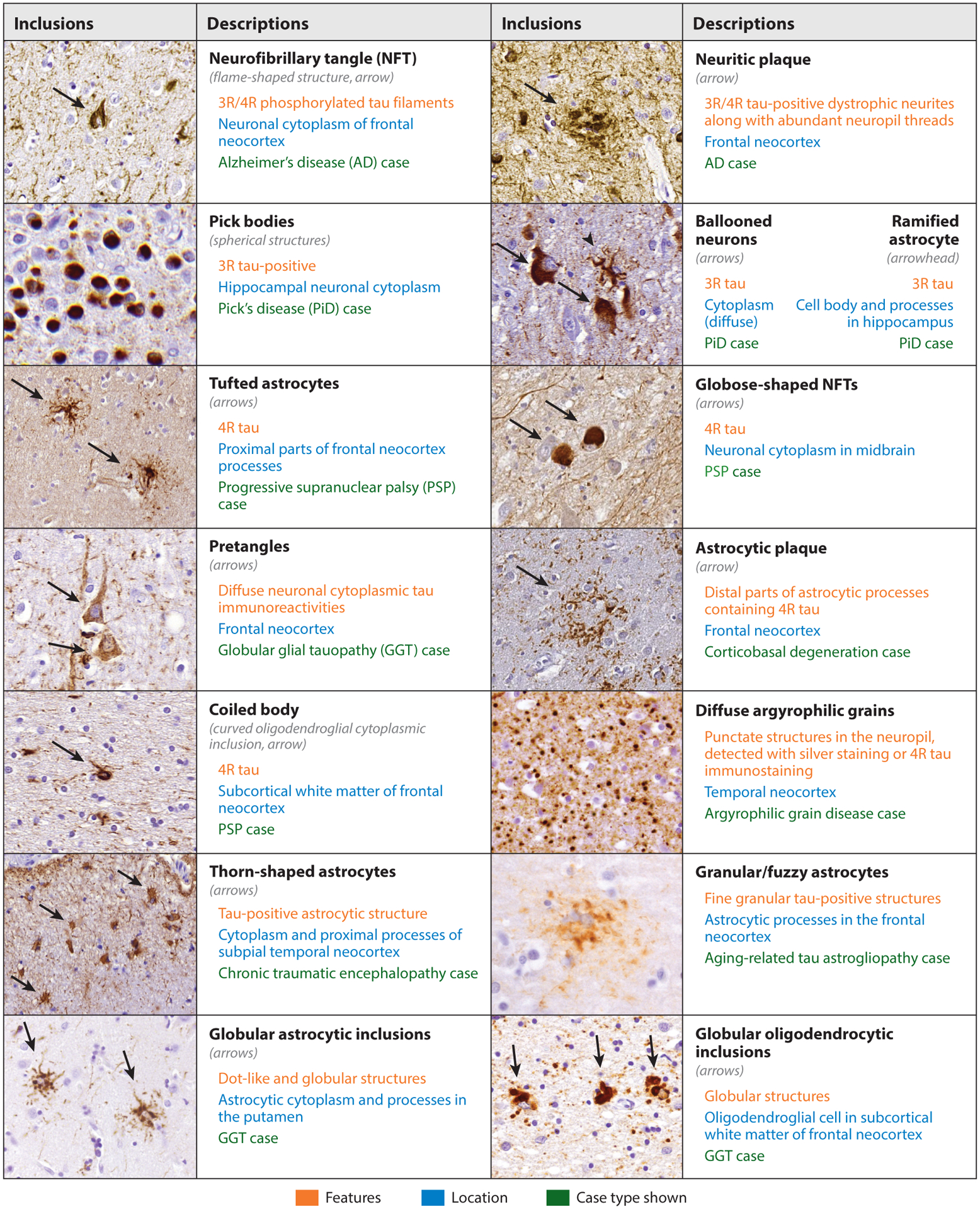
Immunohistochemistry of various tau inclusions, including examples of a neurofibrillary tangle (NFT), Pick body, tufted astrocyte, pretangle, coiled body, thorn-shaped astrocyte, globular astrocytic inclusion, neuritic plaque, ballooned neuron, ramified astrocyte, globose-shaped NFT, astrocytic plaque, argyrophilic grain, granular/fuzzy astrocyte, and globular oligodendrocytic inclusion, found in various tauopathies.

The clinical and pathologic features of tauopathies are diverse, representing a range of cognitive and movement disorders with a variety of tau-positive neuropathologic inclusions (Figure 3). Clinical syndromes are sets of findings that describe similar patient presentations and, in the case of neurodegenerative diseases, typically indicate which region of the nervous system is affected. However, there is an imperfect concordance between clinical syndromes and the underlying disease process such that defined clinical syndromes sometimes but not always predict the underlying neuropathologic change. Most tauopathies cannot be confirmed until autopsy with the notable exceptions of the rare autosomal dominant tauopathies or cases diagnosed clinically with progressive supranuclear palsy, which is highly predictive of a tauopathy (although the subtype of tauopathy cannot always be defined antemortem). Most tauopathy-associated clinical syndromes are defined by dominance of specific signs or symptoms. However, there is considerable heterogeneity such that several different clinical syndrome variants can be associated with the same underlying disease process and clinical phenotypes can change over the course of a disease. Indeed, there has been a movement toward a more biological definition of disease where biomarkers that reflect underlying neuropathologic change are used to augment clinical diagnosis (31), which has traditionally been solely based on clinical syndromes. Here we describe the clinical syndromes associated with underlying tauopathy.

### Alzheimer’s Disease

AD can be the underlying pathology in many different clinical syndromes. AD is commonly characterized clinically by amnestic dementia that includes episodic memory deficits followed by progressive impairment of executive, visual, language, and neuropsychiatric domains. Nonamnestic syndromes include logopenic variant of primary progressive aphasia (PPA), behavioral variant of AD, posterior cortical atrophy, and corticobasal syndrome (CBS). Pathologically, AD is characterized by intracellular NFTs and extracellular Aβ plaques. Other features seen are distortion of neuronal processes with tau present forming dystrophic neurites and neuropil threads.

### Pick’s Disease

The most common clinical syndrome of Pick’s disease (PiD) is behavioral variant of FTD, but it can be found in cases of semantic variant of PPA, nonfluent agrammatic variant of PPA, and CBS. Pathologic hallmarks of PiD are intraneuronal Pick bodies consisting of spherical tau aggregates. Other findings include tau-positive ramified astrocytes and ballooned neurons.

### Progressive Supranuclear Palsy

A common clinical presentation of progressive supranuclear palsy (PSP) is PSP syndrome (PSPS) characterized by an atypical parkinsonian syndrome with axial rigidity and poor dopamine response. PSPS with supranuclear gaze palsy and postural instability are specific for PSP pathology. Other clinical phenotypes seen include nonfluent agrammatic variant of PPA, CBS, and behavioral variant of FTD. Pathologic hallmarks of PSP include tau inclusions in the form of tufted astrocytes, globose tau inclusions in the brain stem and subcortical neurons, NFTs, and oligodendroglial coiled bodies.

### Corticobasal Degeneration

Many cases of corticobasal degeneration (CBD) are associated with CBS clinically. However, CBS is not specific for CBD pathology, and <50% of CBS patients have CBD pathology. CBS is defined by asymmetric parkinsonism, apraxia, executive dysfunction, and parietal lobe dysfunction including sensory neglect. Other clinical phenotypes include PSPS, nonfluent agrammatic variant of PPA, and behavioral variant of FTD. Pathologic hallmarks of CBD include abundant threads in gray and white matter together with astrocytic plaques and ballooned neurons in gray matter of limbic and neocortical regions. Additionally, there are coiled bodies and astrocytic tau inclusions in white matter.

### Argyrophilic Grain Disease

Clinical syndromes are heterogeneous for argyrophilic grain disease (AGD) and range from asymptomatic to a slowly progressive amnestic syndrome. Pathologic hallmarks are spindle-shaped tau-positive argyrophilic grains primarily found in the hippocampus and amygdala. Additional findings are pretangles, coiled bodies, ballooned neurons, and NFTs.

### Globular Glial Tauopathy

The clinical phenotype of globular glial tauopathy (GGT) is varied with no single clinical feature representing more than approximately one in four cases. The most common clinical features are PPA, behavioral variant of FTD, upper motor neuron signs, memory impairment, and PSPS. Pathologic hallmarks of GGT include globular tau inclusions in oligodendrocytes, star-shaped globular astrocytic tau inclusions, coiled bodies, tufted astrocytes, and neuronal tau inclusions.

### Chronic Traumatic Encephalopathy

The clinical features that define traumatic encephalopathy syndrome (TES) have recently been proposed, which include a history of repetitive head impacts together with progressive cognitive impairment or neurobehavioral dysregulation (32). Beyond the history of head injury, TES criteria are nonspecific with many features overlapping with other neurologic and/or psychiatric diseases. Pathologic chronic traumatic encephalopathy (CTE) diagnosis requires tau inclusions at the depths of the cortical sulci around blood vessels; CTE is often a complex lesion consisting of neuronal NFTs but also often containing tau-positive thorn-shaped astrocytes.

## TRANSMISSION

The variability of clinical phenotypes across the tauopathies appears to be related to differences in the distribution of tau aggregates observed for each entity. Thus, AD is characterized by tauopathy in the early stages that localizes to the medial temporal lobe including the hippocampus before spreading to the rest of the cerebrum, which correlates well with the early amnestic clinical phenotype that progresses to involve other cognitive domains. In contrast, PSP is characterized by a preponderance of brain-stem and subcortical involvement, correlating well with an atypical parkinsonism syndrome. Indeed, a growing literature suggests that tau spreads through the human brain from region to region through the neuronal connectome where tau aggregates transmit from one cell to another. Thus, tau aggregates that form in one neuronal compartment appear to spread through the central nervous system in a process where a pathologic protein seed is transferred from an affected cell to a neighboring unaffected cell, corrupting its normal tau protein by a process of seeded nucleation, ultimately resulting in tau fibrils that are neurotoxic. Studying the processes that determine how tau propagates through the neuraxis is critical toward understanding the pathophysiology of these diverse diseases.

Unlike major prion protein (PrP), which misfolds and transmits in the setting of prion disease, tau aggregates have not been shown to be infectious nor to stably propagate (33). Aβ aggregates, the coaggregate of tau in AD, have been proposed to form after iatrogenic transmission through Aβ seeds secondary to human cadaveric pituitary-derived growth hormone treatments or dural grafting (34–36). Tau accumulation has been seen in cases of suspected iatrogenic Aβ seeding, but only after very long incubation times of >35 years where tau accumulation may be a secondary consequence of Aβ accumulation as opposed to direct tau seeding (37).

In the setting of sporadic AD, tau aggregates are thought to initiate in subcortical structures, namely the locus ceruleus, before stereotypical progression of neurofibrillary degeneration into limbic regions, including the transentorhinal cortex and hippocampus, followed by broader neocortical involvement codified as the Braak stages of AD (2, 3). Interestingly, regions of the brain most strongly connected to the locus ceruleus and transentorhinal cortex generally have more tau in AD inferred by tau positron emission tomography (tau-PET) and functional magnetic resonance imaging, supporting a transneuronal spread of tau model for AD (38). In later stages, tau aggregates spread to other associated neocortical regions (frontal, temporal, and parietal) with NFTs being found in the primary visual cortex in the occipital lobe defining the most advanced stages (3). While the distribution of neurofibrillary degeneration is remarkably consistent across the vast majority of AD cases, variations have been described that represent opposite ends of the spectrum of AD neuropathologic change—on the one hand are cases with relatively less involvement of the hippocampus (hippocampal sparing) and on the other hand are cases with relatively less involvement of extrahippocampal regions (limbic predominant) (39). Another neuropathologic variant is cases where tau accumulation is particularly evident in the occipital lobe (40). These histologic variants are often associated with atypical clinical phenotypes such as nonamnestic forms of AD (such as behavioral/dysexecutive variant of AD) in hippocampal sparing AD and posterior cortical atrophy (characterized by visuospatial abnormalities) in cases with prominent occipital involvement. While the biological basis for these atypical AD phenotypes is not known, we recently found that genetic risk variants in *TREM2* are associated with hippocampal sparing and nonamnestic AD (17). Additional work is needed to determine the molecular mechanisms that regulate the spread and distribution of tau in AD and related dementias.

As AD is by far the most common tauopathy, studies on AD dominate our understanding of how tau is transmitted. Neuropathologic staging schemes similar to the AD Braak stages have also been proposed for other tauopathies such as PSP (41), although perhaps with more variability than what is seen in AD. Moreover, mechanisms of tau transmission are likely not the same for all tauopathies. Tau-PET studies have suggested that AD tau is primarily transmitted via direct neuronal connections, while PSP tau generally progresses to areas of higher metabolic demand with less trophic support (38). Whatever the mode of transmission, it appears that tau fibrils are able to induce pathology in adjacent or connected brain regions resulting in the spread of aggregation-related toxicity (42). Even recombinant tau fibrils can seed tau aggregation, recruiting normal tau to make tau conformations that can seed further, albeit with lower efficiency compared with human brain-derived tau seed preparations (43, 44).

Cerebrospinal fluid studies provide evidence that tau can be found extracellularly and is not solely an intracellular protein (45). The possibility that tau transmission involves transit of tau extracellularly makes it an enticing target for immunotherapy whereby antitau antibodies would help clear tau. However, so far, tau antibodies have been ineffective clinically, despite the fact that via biofluid measurements they reduce soluble tau (46, 47). Several groups have shown that soluble tau oligomers are responsible for seeding in different model systems (48–55). However, since many of these species are rare, it is possible that a reduction in soluble tau measured in biofluids may not reflect a reduction in these rare, but important, species. Moreover, many groups have shown that insoluble fractions of tau exhibit robust seeding activity (43, 56–58). Given that solubility does not necessarily infer molecular structure (some soluble oligomers have been shown to exhibit fibrillar ultrastructure) (59), it is not clear what characteristics, including number of tau molecules, structure, and stability, convey seeding capacity. Moreover, it remains possible that there can be multiple seeding competent species. Further understanding of how tau moves between cells in humans and induces new pathology may help the development of effective therapeutics.

One factor that appears to dictate the pattern and potency of transmission may be the intrinsic properties of the tau seeds themselves (58, 60, 61). AD tau, recombinant 2N4R tau (T40) that was fibrillized without cofactors (self-seeded), and T40 that was fibrillized with heparin as a cofactor (heparin-induced) have different seeding abilities. Heparin-induced tau, a fibril commonly used to model disease, showed the lowest seeding activity in nontransgenic mice (43, 62). AD tau, unsurprisingly, exhibits more seeding activity compared with self-seeded T40, where recombinant tau seeds that had been seeded in vitro with human AD tau lysates had intermediate potency (43). The different seeding potencies of different derivations of tau fibrils indicate that fibril conformation may be important for disease propagation. In addition, because human AD tau templated recombinant tau had reduced seeding capacity compared with AD tau itself, PTMs may also play a role in determining seeding potency. Moreover, seeding of recombinant protein using human brain-derived proteopathic seeds can sometimes result in aggregates that do not faithfully recapitulate the original seed conformation (63), and so it is not certain that templating recombinant tau with human AD tau truly recapitulates the same conformation as human AD tau. It is also unclear what PTMs, if any, affect seeding activity, as phosphorylation alone was not enough to improve seeding efficiency (43, 56). The in vivo fibrillization process is likely very different and more iterative than the relatively short, isolated in vitro fibrillization process. In vivo fibrillization may allow back and forth fibril creation and destruction that favors pathogenic and thermally stable conformational variants, where in vitro fibrillization likely results in kinetically favored conformations. Indeed, in vivo seeding, unlike in vitro seeding, appears to better recapitulate the original tau seed properties (discussed further below) (58). That being said, conditions for generating human AD tau conformations using recombinant tau protein have been identified through an empiric and iterative process (64).

Cellular patterning of transmission in addition to potency seems to be inherent to the seed type. In mice, intracerebral injections of pathologic tau show cell-type specificity similar to that of the human disease from which the tau was sourced (43, 58, 61, 65, 66).Injecting tau seeds from AD, CBD, PiD, and PSP into mice showed cell-type specificity and tau isoform specificity that matched that of the original disease even through repetitive in vivo seeding procedures (58). Additionally, PiD seeds composed of only 3R tau could not induce pathology in 4R-only mice, PSP/CBD seeds composed of only 4R tau could not induce pathology in 3R-only mice, and AD seeds composed of 3R+4R tau could induce tau in both 3R-only and 4R-only mice (58). These experiments demonstrate that transmission pattern and even isoform composition are likely encoded within the tau seed itself, likely through maintenance of specific conformation and/or PTMs. Moreover, these two factors may not be wholly independent where different structural conformations may allow for or be stabilized by specific PTMs that could be dictating cell type and perhaps even regional specificity for tau transmission. The downstream clinical phenotypes that differ across tauopathies appear to be dependent on the differential neuronal and regional vulnerabilities specific to each tauopathy, and this may be dictated by the type of tau aggregate that forms early in disease and continues to propagate in a type-specific manner.

In tauopathies, research has historically focused on neurons due to their importance for cognition and the prevalence of pathology seen in these cells. Additionally, for AD, neuronal tau aggregates vastly outweigh glial involvement. However, glia play an important role in proper brain function and likely have distinct mechanisms of tau transmission from neurons. In non-AD tauopathies, glia are often prominently affected and show distinct tau aggregates with biochemical and morphological differences from neuronal and AD tau (6). Interestingly, when neuronal tau was knocked down in mice, oligodendroglial tau aggregates were still able to propagate along white matter tracts, resulting in loss of oligodendroglia (67). In contrast, astrocytic tau aggregates still formed initially in vivo but did not spread over time (67). This result is consistent with the idea that astrocytes may be a sink for pathologic aggregates that could be harnessed to mitigate tau transmission. Increasing transcription factor EB (TFEB), a regulator of lysosomal biogenesis, in astrocytes in mice increased tau fibril uptake via astrocytes and reduced spreading of tau aggregates from the ipsilateral to contralateral hippocampus (68). In addition, autopsy studies of individuals with PSP who were administered antitau passive immunotherapy exhibited tau accumulation within astrocytic lysosomes, suggesting that astrocytes may serve as a sink for pathologic tau (69).

Nonhuman primate studies have provided a link between experimental rodent studies and observational human studies. Using human AD brain homogenates, seeding in long-term marmoset (*Callithrix jacchus*) studies showed only sparce Aβ deposition at 3.5 years postinjection. Even at 7 years postinjection, AD-like features were not reported (70–72), potentially indicating marmoset resistance to AD development or highlighting the drawn-out development of detectable disease. Switching model systems, however, yielded more informative results. Inoculation of mouse lemurs (*Microcebus murinus*) in the hippocampus led to cognitive decline, functional alterations, and cerebral atrophy with neuronal loss in the hippocampus and entorhinal cortex but sparse Aβ and tau deposits in regions near the inoculation site (57). Notably, the animals that were tau positive, not just Aβ positive, had the lowest performance in memory tasks and displayed the greatest neuronal loss (57), highlighting tau’s central role in cognitive decline and neurotoxicity. In follow-up work, sonicated AD tau seeds were injected into the posterior cingulate cortex and corpus callosum of mouse lemurs, leading to widespread tau and Aβ pathology (73). While highlighting that tau extracts can indeed be transmitted into primate brains, the studies seem to support the idea that tau transmission and spread are likely regulated by intrinsic regional susceptibility and not only due to connectivity and/or diffusion. Regions spatially adjacent to the inoculation site were spared, and regions connected to the cingulate cortex, such as the entorhinal cortex, exhibited tau aggregates but were spared of Aβ deposits (73). Although regional susceptibility to protein aggregation is likely a major contributor to the spread of proteinopathy in the brain, regions of human brains from individuals with AD that lack AD neuropathologic change and generally do not develop tau aggregates appear to contain tau species that can competently seed tau in a cell model (74). Unsurprisingly, aging also plays a role in susceptibility to tau seeding. Tau pathology spreads more readily in older mice (43, 75), while newborn mice are more efficient at clearing tau (76). Thus, there are likely many factors that lead to regional and temporal susceptibility to proteinopathy. While the mechanisms that regulate transmission of tauopathies are still being discovered, recent evidence in mouse and primate models points toward inherent characteristics of the tau seeds themselves as determinants of selective cellular and regional vulnerability that defines the heterogeneity across diverse tauopathies.

## TAU STRAINS/STRUCTURAL BIOLOGY

**Figure 4:**
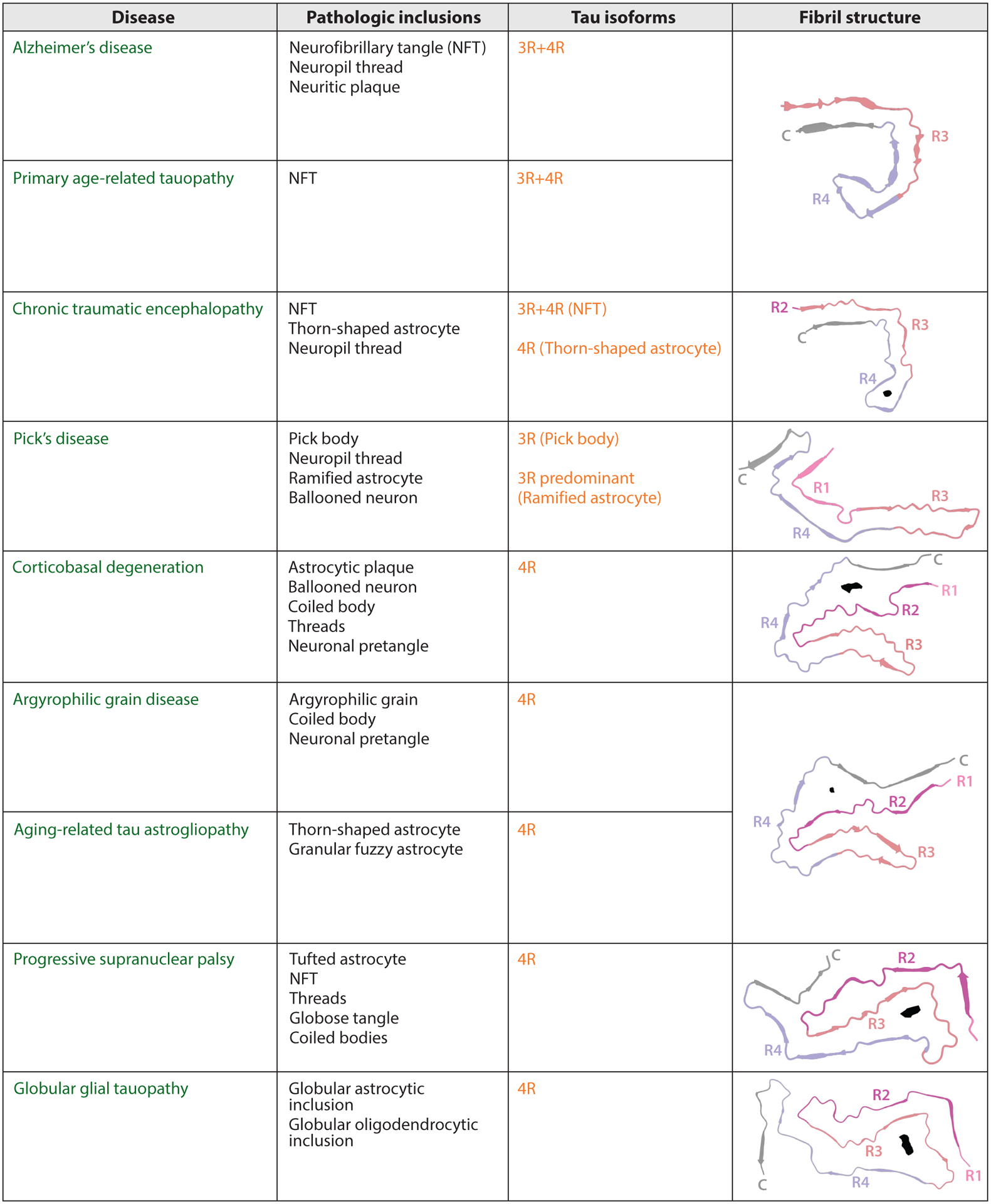
Disease-associated tau fibril cryogenic electron microscopy structures. Diseases with a high-resolution fibril structure are listed with pathologic inclusions found and tau isoform present by immunohistochemistry. Fibril structures are shown for Alzheimer’s disease [Protein Data Bank (PDB) number: 5O3L], primary age-related tauopathy (PDB: 5O3L), chronic traumatic encephalopathy [PDB: 6NWP, Electron Microscopy Data Bank (EMDB) number: 0527], Pick’s disease (PDB: 6GX5), corticobasal degeneration (PDB: 6TJX, EMDB: 10514), argyrophilic grain disease (PDB: 7P6D, EMDB: 13226), aging-related tau astrogliopathy (PDB: 7P6D, EMDB: 13226), progressive supranuclear palsy (PDB: 7P65, EMDB: 13218), and globular glial tauopathy (PDB: 7P67, EMDB: 13220) with the core region colored by tau domain (R1: *light pink*, R2: *dark pink*, R3: *salmon*, R4: *purple*, C-terminal region: *gray*) and other notable internal cavity densities highlighted (*black*). Images created using UCSF ChimeraX, developed by the Resource for Biocomputing, Visualization, and Informatics at the University of California, San Francisco, with support from National Institutes of Health R01-GM129325 and the Office of Cyber Infrastructure and Computational Biology, National Institute of Allergy and Infectious Diseases (79).

Electron microscopy (EM) allows for information at a resolution beyond traditional light microscopy. EM has shown that tau fibrils generally have a structured core surrounded by an unstructured region called the fuzzy coat for its fuzzy appearance by EM that contains the unstructured regions of the tau protein, PTMs of tau, and sometimes other proteins and cofactors that may associate with tau fibrils (77).EM has also shown that for many tauopathies the fibrils are formed by two tau molecules coming together, then stacking in pairs to form long, twisted fibrils made of two distinct filaments (77). The filament interaction can change the shape of the fibrils, leading to distinct ultrastructural polymorphs characterized by changes in helical structure even within the same disease [AD: twisted paired helical filaments (PHFs) versus straighter straight filaments (SFs); CBD: PHF-like type 1 versus tubular type 2 filaments; PiD: straight filaments and twisted ribbons]. In the past decade, advances in cryogenic EM (cryo-EM) have changed the understanding of the underlying molecular structures of different tau strains. Prior to cryo-EM, EM studies allowed for determination of the general shape of the two tau molecules coming together in AD-associated PHFs and SFs (77). Now, cryo-EM allows for near-atomic resolution of fibrils that can be fit with atomic models. Cryo-EM requires small sample sizes and can accommodate more heterogeneity than other high-resolution methods such as X-ray crystallography or solid-state nuclear magnetic resonance. Brain-extracted tau fibrils are not amenable to these other techniques and so our understanding of tau fibril structure has benefitted significantly from advances in cryo-EM. Structures of tau fibrils from at least 15 different diseases have been resolved to high resolution (78), providing insight into the different tau fibril conformations that are associated with different tauopathies (Figure 4). A major hypothesis that integrates neuropathology, clinical, and biochemical concepts is that disease heterogeneity among the tauopathies is encoded in structural variations of tau filaments found in disease, perhaps defining different tau strains that dictate spread through the brain and ultimately clinical phenotypes. The enhanced structural understanding of strains puts into context many years of in vitro and in vivo experiments to better understand factors that mediate the differential effects of tau strains. However, not all strains have been solved to high resolution, and the factors that create and propagate different strains have not been fully identified. For example, the fuzzy coat that can be seen by EM lacks enough regular structure to identify PTMs, cofactors, and other proteins that may be contained there, as cryo-EM relies on averaging the same structural organization many times.

Strains appear to retain their seeding potency after in vivo propagation (58) but not in vitro propagation (43, 80). However, this discrepancy may be protocol specific (64) or reflect the fact that in vitro amplification does not recapitulate the structure of the original seed (63). Biochemical studies and light microscopy showed that tau inclusions in mice have similar properties to the inclusions of the original disease from which the tau strain was taken (58). Moreover, seeding T40 (2N4R) recombinant tau with human brain extracts in vitro retained strain-dependent characteristics when the recombinant fibrils were used to induce tauopathy in mice, but at a reduced potency. Morphologically, the T40 amplified with AD tau looked similar to the original AD tau by negative-stain EM, but high-resolution cryo-EM could confirm if this amplified tau has the same fibril structure as AD tau. These T40 fibril preparations that were amplified with AD tau extracts in vitro appeared to retain strain-like properties when used to induce tauopathy in mice, as they developed tau aggregates containing equal ratios of 3R and 4R tau, similar to the AD tau seed (80). Therefore, even if the fibril structure was altered, it was at least similar enough not to exclude either 3R or 4R tau from being incorporated into tau aggregates. That being said, protocols whereby recombinant tau can be coaxed into forming the same fibril core structure as that seen in human AD tau fibrils have been developed by Lövestam et al. (64), albeit requiring a truncated version of tau that lacks the complexity of the full-length protein including various PTMs present in tau filaments extracted from AD brain. Cellular seeding experiments, however, were able to mostly recapitulate AD tau fibrils with full length that likely have at least some PTMs (81). Future experiments could shed light on the importance of structure alone if the PHFs generated by Lövestam et al. (64) could induce a similar effect in mice to the T40 amplified by brain extracts. If the seeding characteristics are similar, then structure of the tau fibril core is indeed more important than PTMs. The recombinant fibrils generated by Lövestam et al. (64) specifically mimic AD PHFs, but the principle of amplification of recombinant tau using human brain extracts, shown by Xu et al. (80), held true for PSP and CBD tau as well, indicating that seeded amplification and strain-like behaviors are not AD-only phenomena.

With the recent publication of cryo-EM structures from 15 different tauopathies (Figure 4), the granularity of similarities and differences between different disease-associated fibrils has been greatly increased and, in some instances, provides insights into biological processes previously observed. For example, AD tau can template either 3R or 4R recombinant tau, whereas brain-derived tau filaments from 3R-only or 4R-only tauopathies cannot necessarily recruit the other isoform (43). The cryo-EM structures of AD tau provides a plausible explanation, as the AD tau fibril core mostly incorporates only the third and fourth repeat domains of the MTBR, which would allow for templating of either 3R or 4R tau protein (78). In contrast, the fibril core in PiD, a 3R-only tauopathy, includes the first repeat domain directly connected to the third repeat domain, preventing compatibility with a 4R tau protein that contains the second repeat domain as well (82). Additionally, all known 4R tauopathy fibril cores include the second repeat domain that is not present in 3R, preventing compatibility with 3R tau (78). These structures provide a structural basis for isoform-dependent tau seeding.

There are several ways to categorize tauopathies on the basis of their clinical phenotypes, microscopic pathology, and protein biochemistry. The advent of cryo-EM-based tau filament structures now allows for a structural classification of tauopathies. Generally, the 3R/4R tauopathies have a similar fold, with sporadic AD, familial British dementia, familial Danish dementia, primary age-related tauopathy, and AD due to p.V717F *APP* all exhibiting a similar fold (78, 83). In contrast, CTE shows a more open fold that contains a hydrophobic pocket with an unknown nonproteinaceous density (84). Both CTE and AD fibrils have been found in other diseases in appreciable amounts, including ~47% of fibrils in familial British dementia being the CTE fold and 62% of fibrils in an AGD case being the AD fold (78). These crossovers of fibril structure may indicate that no single structure can explain every disease or the predominance of copathologies, as the patient with familial British dementia was noted to have a history of traumatic brain injury (78). For the 4R tauopathies, the folds can be subdivided into four-layer versus three-layer folds (78). The four-layer folds from cases of CBD, AGD, *MAPT* intron 10 +3 and +16 mutations, and aging-related tau astrogliopathy have very similar structures, with CBD having a relatively distinct fold (20, 78, 85). The main difference between the folds is a larger, more positively charged cavity with nonproteinaceous density in CBD compared with the other four-layer folds (20, 78, 85). For the three-layer folds, the structures are again quite similar with notable distinctions lying in the size and charge of the largest cavity, which is larger and more positively charged in GGT and in PSP (78). While the different structural folds have been hypothesized to underlie different tau strain behaviors, neuropathologic diversity, and clinical phenotypes, additional work is still required to fully understand the significance of the structure-based classification of disease. For example, there is heterogeneity in terms of how much these entities overlap with regard to microscopic neuropathology and clinical symptomatology within the 4R tauopathies. Thus, although some 4R tauopathies, most notably CBD and PSP, share some clinical and pathologic phenotypes, they have strikingly different fibril folds (86).

For the most part, distinct tauopathies have distinct near-atomic resolution fibril structures (78). A logical question is whether the structure of the fibril core itself is the distinguishing factor of strains’ different biological effects or whether PTMs, cofactors, or other bound molecules that are compatible with only certain structures are the distinguishing factor. Indeed, some structures also have nonproteinaceous molecules or unexplained cryo-EM density inside or outside the core structure that may stabilize a given fibril type and, if identified, could provide insight into factors that regulate the fibrillization process (20, 78). One hypothesis is that these densities could represent sequestered molecules whose sequestration may contribute to neurotoxicity (87). In the case of CBD, there is an internal cavity that likely contains a negatively charged small molecule that connects to lysine (K290 and K370, also possibly K281 and K294) (20), whereas other internal cavities, such as one in the PSP fold, likely contain hydrophobic molecules (78). While one can speculate about the identity of these small molecules on the basis of in vitro experiments for tau fibril formation, it will be difficult to identify these molecules in vivo or ex vivo, as they likely cannot be resolved via cryo-EM or other currently available methods. However, in vitro fibrillization that formed CTE-type fibrils showed a similar hydrophobic cavity with nonproteinaceous density (64). With very few reagents, it is possible that this density formed in vitro is from dithiothreitol, sodium chloride, or even sodium phosphate (64). Whether this indicates the same identity of the in vivo CTE-type fibrils is unknown. It may also indicate that the nonproteinaceous densities do not always have the same identity and rather represent a collection of molecules that share similar properties important to fibrillization. Interestingly, using CBD tau to seed aggregation in a cell model yielded CBD-like fibrils that notably lacked the ordered internal cavity containing nonproteinaceous density (81), further highlighting there are still many unknowns as to how fibrils form their different structures and what the role of nonproteinaceous densities is. Many fibrils have densities external to the fibril core as well. Cryo-EM structures of AD and CBD fibrils have extra densities around external lysines. The identity of the densities is difficult to ascertain by cryo-EM due to these densities likely having a different periodicity than the fibril itself or minimal repeating structure. The location near external lysines, however, may suggest that ubiquitin or other molecules prone to associate with lysine, such as RNA, are stabilized between different levels of the fibril, thus showing density via cryo-EM (20). These molecules may have a role in stabilization of fibrils or may be one factor that causes the very specific tau fibril conformations found in human disease that have been difficult to recapitulate in vitro. It has been shown that RNA is sequestered in NFTs in a variety of tauopathies including AD, PSP, CBD, PiD, and amyotrophic lateral sclerosis and parkinsonism-dementia complex of Guam and Pick bodies from PiD (88, 89). The sequestered RNA is likely within the fuzzy coat and, if it indeed binds to external lysines on the ordered core, could contribute to the extra density seen in cryo-EM reconstruction tau. Tau has a propensity to interact with polyanions such as RNA. Polyanions can compete for tau binding to tubulin and likely can inhibit microtubule assembly in a sequence agnostic way (90). In vitro, the addition of various polyanions, including RNA, induces the fibrillization of tau (91, 92). Additionally, molecular crowding or the addition of RNA at physiologic buffer conditions induced tau condensates that were able to seed tau aggregation and nucleocytoplasmic transport defects in cells (93). Interestingly, soluble, but not insoluble, seeds isolated from AD brains have reduced seeding capacity upon ribonuclease treatment (94), potentially indicating that RNA has a role in the initial stabilization of tau aggregates but is less important once stable aggregates are formed.

With regard to PTMs, not all modifications are compatible with all fibrillar structures, and it has been suggested that, even within AD and CBD, the different fibril types (SF versus PHF for AD) may be explained by varied acetylation or ubiquitination at specific sites (20). Indeed, AD tau fibril acetylation and ubiquitination primarily occur in the MTBR or in the adjacent C-terminal region (Figure 2) that are part of every known disease-relevant tau fibril core (22, 78). However, many other PTMs localize to the tau filament’s fuzzy coat, which is relatively unstructured. Through iterative and empiric attempts to create AD tau filament core structures from recombinant tau, subtle changes in molecular environment can drastically change fibril structure (64). Many conformations of tau fibrils are possible and have been seen in vitro. Thus, it is interesting that only a select few conformations are found in human disease (64).

Even with all these high-resolution data, it is still unknown what species are necessary or sufficient for seeding of tau filaments in vivo. It is possible that cryo-EM methods have identified only the major species present in human tissues and that there are rare, but biologically significant, species that have not been identified. Cryo-EM would suggest that species in the same insoluble fraction have not been missed since there have been relatively few reported low-abundance species that do not resolve to high resolution; even those that do not allow for atomic modeling resemble atomically modeled structures (78). Cryo-EM fibril samples have generally been particle-picked manually, so cryo-EM is unlikely to miss rare fibrils present in the sample. Sample preparation, however, may introduce biases resulting in the loss of certain fibril types. For example, in the search for TDP-43 fibrils that form in the setting of FTD, some biochemical fractions contained TDP-43 filaments while other fractions yielded a distinct fibril composed of TMEM106B protein (95, 96). Another example is CTE, where 3R+4R neuronal inclusions are found in the same lesion at the depth of sulci as 4R-only glial inclusions (10). Presumably due to the methods used to extract insoluble tau filaments, only 3R+4R tau filaments have been solved by cryo-EM (78, 84). Indeed, large mature tau fibrils seem unlikely to be the main disease-relevant seeds due to their size. Large mature fibrils are likely too large for cell-to-cell transportation. The minimum number of tau units sufficient for seeding competence, however, is unclear. There is competing evidence as to the minimum number of tau molecules necessary to seed fibrils, with some evidence saying even one misfolded tau monomer is sufficient (48, 52–54). It is also possible that the size of the tau seed may not matter, but rather its structure is the most important factor for seeding competence (53, 54). Nevertheless, the properties that confer seeding competence are likely different for the different tauopathies but are somehow encoded within tau filaments, where different strain activities are likely dependent on, at least in part, their varied core filament structures (58, 80, 97).

Cryo-EM has allowed for a detailed structural understanding of how tau forms fibril structures and how these filaments appear. The next steps will be to correlate structure and function to contextualize how these specific structures play or do not play a role in the different tauopathies. Additionally, it will be important going forward to understand how new strains, generated de novo or amplified from human brain filaments, compare with the structures found in disease at high resolution. Finally, a key step will be to visualize tau filaments at high resolution in their natural setting in situ to better ascertain the structural and functional effects that tau filaments have on neuronal function.

## PROTEOSTASIS

**Figure 5:**
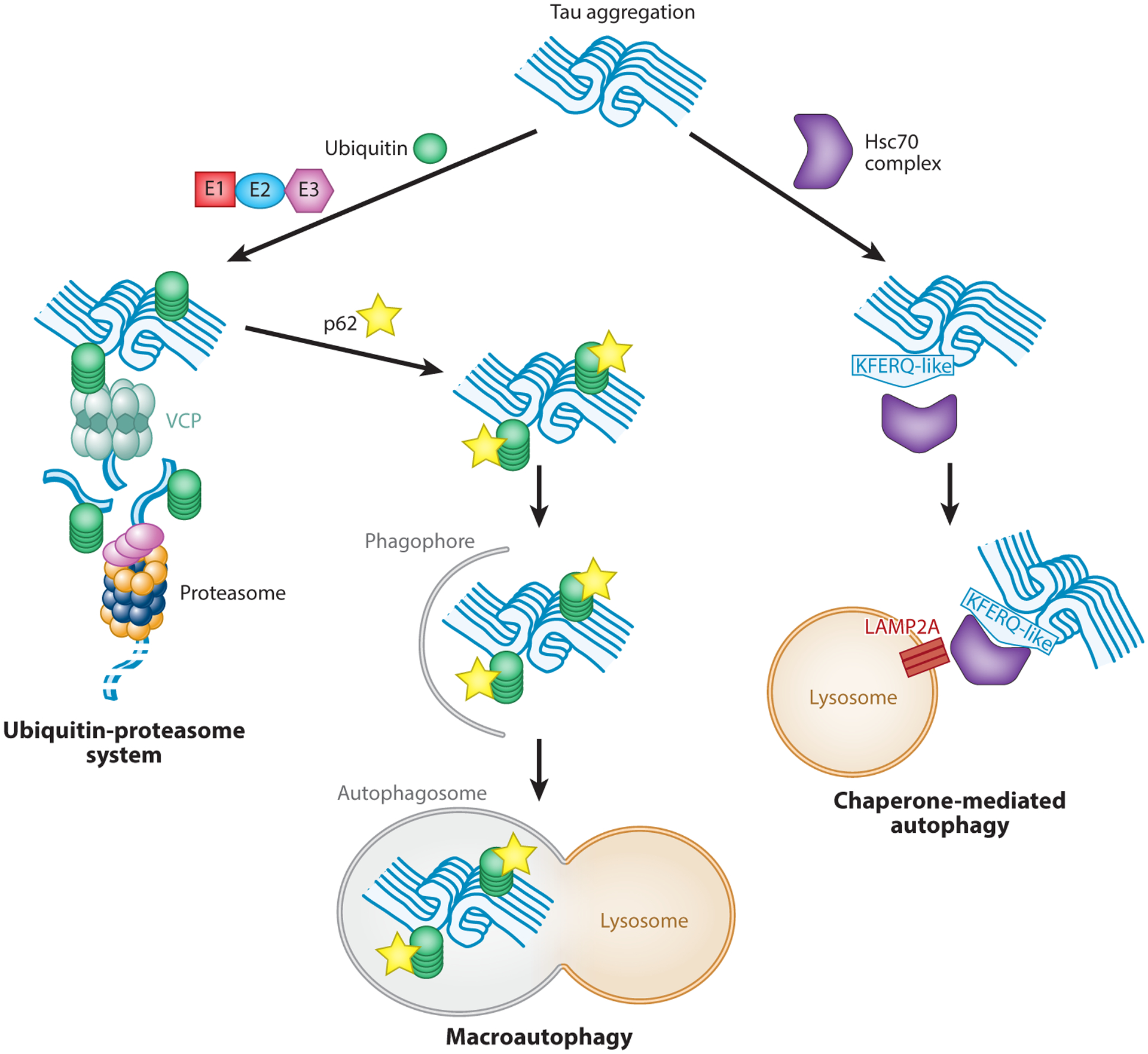
Schematic of tau aggregate clearance pathways. For the ubiquitin-proteasome system (UPS) and macroautophagy, ubiquitin is added to aggregates via a cascade of ubiquitin-activating enzymes (E1s) and ubiquitin-conjugating enzymes (E2s), culminating in a transfer of ubiquitin to an aggregate mediated by an E3 ubiquitin ligase. (*Left*) For the UPS pathway, components of the aggregate are either directly degraded by the proteasome or a protein such as valosin-containing protein (VCP) unfolds and removes a component of the aggregate that is then degraded via the proteasome. (*Center*) For macroautophagy, p62 delivers ubiquitinated aggregates to a phagophore that becomes an autophagosome and ultimately merges with a lysosome for aggregate degradation. (*Right*) Chaperone-mediated autophagy is ubiquitin independent and involves the heat shock cognate 70 (Hsc70) complex binding to a KFERQ-like motif. The Hsc70 complex is then targeted to the receptor lysosome-associated membrane protein type 2A (LAMP2A) on a lysosome for internalization and degradation of the aggregate. Figure adapted from Reference 13.

Protein quality control in cells is generally regulated via two systems, the ubiquitin-proteasome system (UPS) and the autophagy-lysosome system (A-LS) (Figure 5). Rather than ensuring normal protein turnover, dysfunction of these pathways is implicated in diseases characterized by the accumulation of misfolded or aggregated proteins. Dysregulation of these quality control pathways is likely contributing to tauopathy pathogenesis, as exemplified by autosomal dominant pathogenic variants in VCP, a key proteostasis factor, which causes vacuolar tauopathy (13). The UPS is the primary mechanism of degradation of nonaggregated proteins via ubiquitination of target substrates. These target proteins can be degraded directly by the proteasome if there is a flexible region of amino acids (98). In contrast, structured substrates often need to be unfolded by an unfoldase, such as VCP, prior to degradation by the proteasome (Figure 5) (99, 100). The A-LS pathway is more capable than the UPS of degrading structured substrates (101, 102). The A-LS can be divided into macroautophagy, chaperone-mediated autophagy (CMA), and microautophagy pathways. Macroautophagy substrates are tagged by adaptor proteins to target substrates to phagophores. Phagophore membranes form autophagosomes that subsequently fuse with lysosomes, whereupon acid proteases are able to break down proteins (Figure 5) (103). CMA is initiated by the heat shock cognate 70 (Hsc70) chaperone that recognizes a KFERQ peptide motif to target substrates to lysosomes (Figure 5) (104). Protein clearance pathways appear to decrease with age, affecting both the UPS and the A-LS (105). This decrease in protein clearance over time is thought to be a contributing factor underlying the age dependence of neurodegenerative diseases.

Tau aggregation has been associated with many different aspects of proteostasis. Tau aggregates from human brain have been physically associated with proteasome subunits, and proteasomes isolated from AD brain showed an almost 50% decrease in activity compared with age-matched controls that could not be attributed to protein levels (106, 107). Additionally, in vitro and cellular studies have shown that inhibiting the proteasome delays the degradation of tau (108) and activation enhances aggregation clearance (109), highlighting the proteasome’s important role in the degradation of tau and tau aggregates. Ubiquitination, a cellular mark for degradation and a signal for proteasomal degradation, has been shown to be increased in AD tau fibrils, primarily in the ordered MTBR core (Figure 2) (22). Ubiquitination is almost nonexistent by mass spectrometry in tau from cognitively normal individuals (Figure 2) (22). Recent studies have suggested that highly stable ubiquitinated disease aggregates may sequester the proteasome, decreasing its concentration and activity elsewhere in the cell (110, 111). Aggregates not only sequester the proteasome itself but also sequester other key proteostasis components such as Hsc70, Hsp90, and histone deacetylase 6 (HDAC6) (112).

Although the UPS accounts for the vast majority of normal protein degradation, the proteasome has little activity against structured substrates. Thus, aggregates and proteins too large for the proteasome are degraded via autophagy (101, 102). Macroautophagy, as opposed to CMA, is negatively regulated by mammalian target of rapamycin (mTOR). mTOR activation has been shown to increase tau phosphorylation and enhance tau accumulation (113), while inhibition of mTOR with rapamycin improved tau clearance and reduced insoluble tau (114, 115). Induction of macroautophagy independent of mTOR with trehalose also shows improvement in tau clearance (116). Furthermore, AD brains show an accumulation of prelysosomal vesicles, indicating both that macroautophagy may be involved in the clearance of tau aggregates and that this process is impaired in AD (117). Another example of impairment is the decrease of beclin-1, a key component in macroautophagy, in AD brains and the colocalization or sequestration of cleaved beclin-1 (118). The exact defect that leads to impaired macroautophagy, however, remains unclear as macroautophagy is suggested to be increased even in early stages of disease (119). It is possible that macroautophagy is eventually overwhelmed or exhausted in late stages of disease, but there are likely multiple steps of the pathway affected in tauopathies.

Chaperone-dependent pathways in general are impaired by tau aggregation and can be ameliorated with induction of cytosolic chaperone expression by heat shock transcription factor 1 (HSF1) (112, 120). HSF1 increases heat shock protein expression, including Hsc70, to increase proteins brought to lysosomes. HSF1 itself, however, is activated when HDAC6 binds to tau (121). Hsc70, a key component of chaperone-mediated autophagy, recognizes KFERQ peptide motifs to guide proteins to lysosomal degradation. Tau has two KFERQ-like motifs in the MTBR that form part of the aggregate core (78, 122). Since the KFERQ-like motifs are positioned within the structured tau fibril core, Hsc70 may not be able to recognize and efficiently process tau as a substrate. In cellular models, Hsc70 and other chaperones promote tau solubility and microtubule binding for normal tau function (123). Once tau aggregation has begun, however, Hsc70 may be pulled and sequestered into aggregates, similar to what is seen with the proteasome. Conversely, Hsc70 may lose its ability to bind tau since acetylation and phosphorylation, PTMs that promote tau aggregation, also weaken Hsc70 protein recognition and reduce tau degradation by chaperone-mediated autophagy in cell models (120, 124). Mass spectrometry analysis of AD and CBD tau fibrils does not show significant phosphorylation or acetylation on the KFERQ-like motifs of tau directly (Figures 2 and 5), but acetylation and phosphorylation of spatially adjacent amino acids may play a role in blocking the Hsc70 binding site (20, 22).

HDAC6 expression has been shown to be increased in AD where it interacts with tau (125), coaggregating with tau (126). Proteasome inhibition is one signal for HDAC6 to be recruited to polyubiquitinated substrates and be transported to aggresomes for aggregate degradation (127). In cellular models, proteasome inhibition led to an increase in tau-HDAC6 colocalization (125). As aforementioned, the proteasome has decreased activity in AD brains (106, 107) so the cellular state in tauopathies may be similar to that seen upon proteasome inhibition in cell models leading to the recruitment of HDAC6.

HDAC6 deacetylation activity is required for proper aggresome formation (127). HDAC6 likely recognizes polyubiquitin chains on tau fibrils that are physically adjacent to acetylation sites or even occur on the same amino acid in the MTBR (Figure 2) (20, 22). Structural studies of tau fibrils suggest that these sites in the MTBR are likely difficult to access and may even have ubiquitin structuring between many tau monomers (20). If HDAC6 is able to recognize ubiquitin on tau fibrils but not actually deacetylate tau fibrils, this could lead to a defect in the aggresome pathway that is already attempting to compensate for the impaired proteasome pathway. Even if HDAC6 can deacetylate tau, it may be sequestered by tau fibrils, similar to what may be seen with the proteasome and Hsc70 (110–112). With HDAC6 sequestration, clearance of aggregates may be impaired, as HDAC6 depletion accelerates disease in mouse models (126). Additionally, HDAC6-regulated acetylation sites have been described as disease-specific markers for 3R/4R tauopathies and 3R tauopathies (126), which may indicate some differences in PTMs and even aggregation propensity of tau among different tauopathies. In vitro, a K311 acetylation mimic form of recombinant tau aggregated with similar kinetics to P301L tau, a pathogenic tau variant known to have increased propensity for aggregation (126). These data suggest that distinct PTM profiles may play a role in formation of different tauopathies. For example, K311 acetylation is likely more compatible with the PiD and AD tau fold than with the PSP and CBD tau fold (20, 78). Distinct PTM profiles may convey distinct sequestration capacities for different key proteostasis proteins, which may be one factor in distinct disease phenotypes.

VCP is another proteostasis factor important in many quality control pathways, including autophagy and the UPS, and is an essential gene that is required for cellular viability (128–130). When an autophagosome reaches a lysosome, a segregase such as VCP will remove HDAC6 for proper fusion of the autophagosome and lysosome. It is possible that the removal of HDAC6 in this fusion event is impaired, as impairment of VCP function has been associated with tau accumulation (13). Generally, VCP uses ATP hydrolysis to unfold or segregate proteins. Loss of VCP or of its ATPase activity has been linked with the accumulation of autophagosomes and autophagosome components in cellular and mouse models (128). In the UPS pathway, because the proteasome does not efficiently degrade structured proteins, VCP with cofactors recognizes and unfolds structured, polyubiquitinated substrates so that they can be degraded by the proteasome (131). In AD, VCP shows localization to NFTs (13), raising the possibility that VCP, like other proteostasis factors, is sequestered by tau aggregates, leading to a functional VCP deficit. In addition, while the proteasome may get stuck on tau fibrils, in vitro experiments with purified AD tau fibrils suggest that VCP is able to disaggregate tau fibrils (13), which would appear to be beneficial in terms of tau clearance. However, recent cellular data suggest that VCP may actually fragment tau fibrils to generate smaller seeding competent fragments, which could in theory be detrimental if this results in enhanced cell-to-cell transmission (132). In vivo, there may be a bottleneck in the proteasome pathway where VCP is able to fulfill its role upstream from the proteasome, but due to proteasome inhibition, there is not an effective amount of flux through the proteasome pathway. Alternatively, VCP’s colocalization with NFTs may suggest that it too is being sequestered by tau fibrils, leading to a loss of VCP function. Loss of VCP function in the cell would have significant effects on the many pathways it is involved in (130).

While ubiquitin is generally a signal for degradation, in the case of tauopathies it may also paradoxically act to worsen disease by stabilizing and promoting aggregation (20, 29), sequestering HDAC6 and other components of the autophagy pathway (112, 125–127), sequestering or inhibiting the proteasome (106, 107, 110, 111), or sequestering or overrecruiting VCP (13, 132). Supporting this overall pattern, it was shown that using an inhibitor of CHIP, an E3 ligase that ubiquitinates proteins, delayed tau aggregation in vitro and in cell models (29). Overall, however, the dysfunction seen in tauopathies seems to be a failure of multiple different proteostasis modalities that are potentially all marked by the sequestration of key components by tau aggregates. The dysfunction of multiple proteostasis modalities provides opportunities in that there may be several unique drug targets for the treatment of neurodegenerative disease, in contrast with ongoing efforts that have focused on antibody therapy or small molecules that directly disrupt protein aggregates themselves. However, the complexity of the pathways and aforementioned paradoxes makes it difficult to determine if activation or inhibition of given targets is most beneficial.

## CONCLUSION

The tauopathies are a clinically and pathologically diverse group of diseases characterized by the aberrant aggregation of the protein tau. Despite their diversity, however, they are likely connected through underlying mechanisms that dictate how these diseases manifest. Neurodegeneration in the tauopathies is likely associated with propagation of tau aggregates via transmission of distinct tau species that seed new aggregates in connected neurons. Transmission and seeding have been proposed to be a function of the ultrastructure and potentially post-translational modifications of tau aggregates. These tau aggregates disrupt normal cellular function through abnormal interactions with diverse cellular networks, including the proteostasis network. While proteostasis pathways evolved to inhibit aggregate accumulation and therefore transmission, tauopathies have been associated with dysfunction of several proteostasis factors. Thus, revealing the fundamental mechanisms that result in tau accumulation, with a focus on human tissue studies to remain faithful to the real disease, provides hope that targeted, mechanism-based therapies that block tau transmission and enhance proteostasis can be developed for these currently incurable diseases.

## Figures and Tables

**Table 1:** Neurodegenerative disease tauopathies

Group	Tau isoform(s)	Disease(s) (and associated genes)
Autosomal dominant tauopathies	3R or 4R or 3R+4R	*MAPT*-related frontotemporal dementia (*MAPT*)
	3R+4R	Vacuolar tauopathy (VT) (*VCP*)
Sporadic tauopathies^a^	3R	Pick’s disease (PiD)
	4R	Progressive supranuclear palsy (PSP) Corticobasal degeneration (CBD) Argyrophilic grain disease (AGD) Globular glial tauopathy (GGT) Aging-related tau astrogliopathy (ARTAG) Tauopathy with hippocampal 4-repeat tau immunoreactive spherical inclusions
	3R+4R	Primary age-related tauopathy (PART) Amyotrophic lateral sclerosis and parkinsonism-dementia complex (ALS/PDC) of Guam or Kii Diffuse neurofibrillary tangles with calcification, Kosaka-Shibayama disease (DNTC) Astroglial predominant tauopathy Postencephalitic parkinsonism Subacute sclerosing panencephalitis Nodding syndrome
	3R+4R and 4R	Chronic traumatic encephalopathy (CTE)
	3R+4R or 4R	Progressive ataxia and palatal tremor
Autosomal dominant or recessive diseases with multiple pathologies including tauopathy	3R+4R	Alzheimer’s disease (APP, *PSEN1*, *PSEN2*) Down’s syndrome related Alzheimer’s disease (trisomy 21) Prion protein cerebral amyloidosis (*PRNP*) Familial British dementia (*ITM2B*) Familial Danish dementia (*ITM2B*) Niemann-Pick disease type C (*NPC1, NPC2*)
	3R	Myotonic dystrophy type 1 and 2 (*DMPK*, *ZNF9*)
Sporadic tauopathy with β-amyloidosis	3R+4R	Alzheimer’s disease

a Sporadic refers to the lack of known autosomal dominant or recessive genetic cause of disease. Some of these entities have been associated with infectious, environmental (head trauma for CTE), or geographic (Western Pacific for ALS/PCD) factors or may be associated with a family history of disease in some cases (astroglial predominant tauopathy).
